# Supplementary material for: Interactive and unimodal relationships between plant biomass, abiotic factors, and plant diversity in global grasslands
Source: Commun Biol. 2025 Jan 21;8:97. doi: 10.1038/s42003-025-07518-w (PMC11751326; doi:10.1038/s42003-025-07518-w)
Supplement: Supplementary file 2 — Supplement [file 42003_2025_7518_MOESM2_ESM.pdf]

Complex relationships between plant biomass, abiotic factors, and plant diversity in global grasslands

Supplement

Marie Spohn[1*], Sumanta Bagchi[2], Jonathan D. Bakker[3], Elizabeth T. Borer[4], Clinton Carbutt[5,6], Jane A. Catford[7,8,9], Christopher R. Dickman[10], Nico Eisenhauer[11,12], Anu Eskelinen[11,13], Nicole Hagenah[14], Yann Hautier[15], Sally E. Koerner[16], Kimberly J. Komatsu[16], Lauri Laanisto[17], Ylva Lekberg[18], Jason P. Martina[19], Holly Martinson[20], Meelis Pärtel[21], Pablo L. Peri[22], Anita C. Risch[23], Nicholas G. Smith[24], Carly Stevens[25], G.F. (Ciska) Veen[26], Risto Virtanen[13], Laura Yahdjian[27], Alyssa L. Young[16], Hillary S. Young[28], Eric W. Seabloom[4]

[1]Dept of Soil and Environment, Swedish University of Agricultural Sciences (SLU), Lennart Hjelms väg 9, 75007 Uppsala, Sweden
[2]Centre for Ecological Sciences, Indian Institute of Science, Bangalore 560012, India
[3]School of Environmental and Forest Sciences, University of Washington, Seattle, WA, USA
[4]Dept of Ecology, Evolution, and Behavior, University of Minnesota, St Paul, MN, USA
[5]Scientific Services, Ezemvelo KZN Wildlife, Cascades 3202, South Africa
[6]School of Life Sciences, University of KwaZulu-Natal, Scottsville 3209, South Africa
[7]Dept of Geography, King's College London, 30 Aldwych, London, WC2B 4BG, UK
[8]School of Agriculture, Food & Ecosystem Sciences, University of Melbourne, Vic 3010, Australia
[9]Fenner School of Environment & Society, The Australian National University, Canberra, ACT 2600, Australia
[10]School of Life and Environmental Sciences, The University of Sydney, Sydney, NSW 2006, Australia
[11]German Centre for Integrative Biodiversity Research (iDiv) Halle-Jena-Leipzig, Puschstraße 4, 04103 Leipzig, Germany
[12]Leipzig University, Institute of Biology, Puschstraße 4, 04103 Leipzig, Germany
[13]Ecology & Genetics, University of Oulu, PO Box 3000, 90014 University of Oulu, Finland
[14]Mammal Research Institute, Dept of Zoology & Entomology, University of Pretoria, Pretoria, South Africa
[15]Ecology and Biodiversity Group, Dept of Biology, Utrecht University, Padualaan 8, 3584 CH Utrecht, The Netherlands
[16]Dept of Biology, University of North Carolina Greensboro, Greensboro, NC, USA
[17]Dept of Biodiversity and Nature Tourism, Estonian University of Life Sciences, Kreutzwaldi St. 5, 51006, Tartu, Estonia
[18]MPG Ranch and University of Montana, Montana, USA
[19]Dept of Biology, Texas State University, San Marcos, TX 78666, USA
[20]Dept of Biology, McDaniel College, Westminster, MD 21157, USA
[21]Institute of Ecology and Earth Sciences, University of Tartu, J. Liivi 2, Tartu 50409, Estonia
[22]National Institute of Agricultural Technology (INTA), Rio Gallegos, Santa Cruz, Argentina
[23]Swiss Federal Institute for Forest, Snow and Landscape Research WSL, Zuercherstrasse 111, 8903 Birmensdorf, Switzerland
[24]Dept of Biological Sciences, Texas Tech University, 2901 MAin St., Lubbock, TX 79409, USA
[25]Lancaster Environment Centre, Lancaster University, Lancaster, LA1 4YQ, UK
[26]Dept of Terrestrial Ecology, Netherlands Institute of Ecology, Droevendaalsesteeg 10, 6708 PB, Wageningen, the Netherlands
[27]Instituto de Investigaciones Fisiológicas y Ecológicas Vinculadas a la Agricultura (IFEVA), CONICET, Faculty of Agronomy, University of Buenos Aires, Argentina
[28]Dept Ecology, Evolution, and Marine Biology, University of California Santa Barbara, CA USA

*Corresponding author. Email: marie.spohn@slu.se

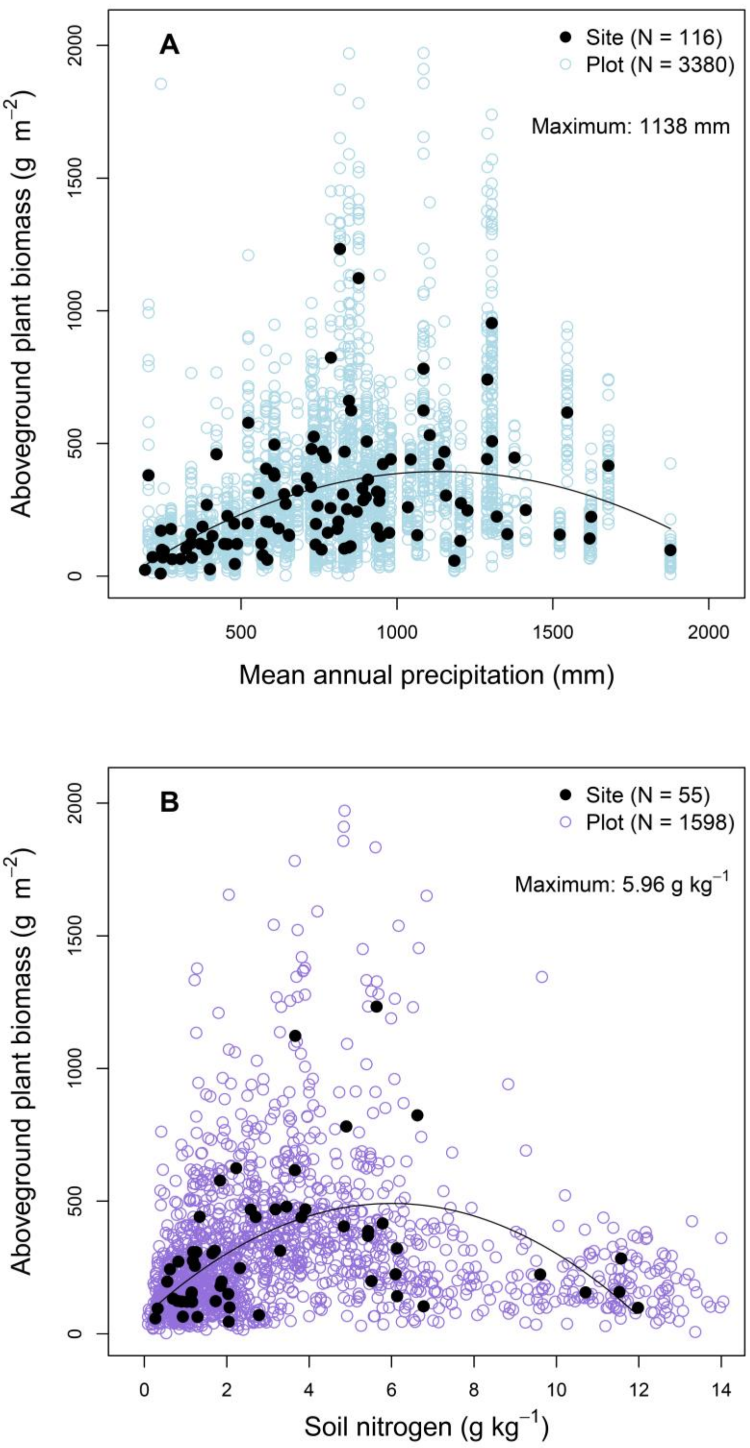

Figure S1 Aboveground plant biomass (APB) as a function of mean annual precipitation and soil total nitrogen. Precipitation ranges from 192 to 1877 mm (N = 116; A) and the soil total nitrogen content ranges from a site mean of 0.2 to 12.0 g kg^{-1} (N = 55; B). The quadratic models were calculated based on the site-level data (and not the plot-level data, which are shown to give insight into the variability). The maximum was calculated based on the quadratic model.

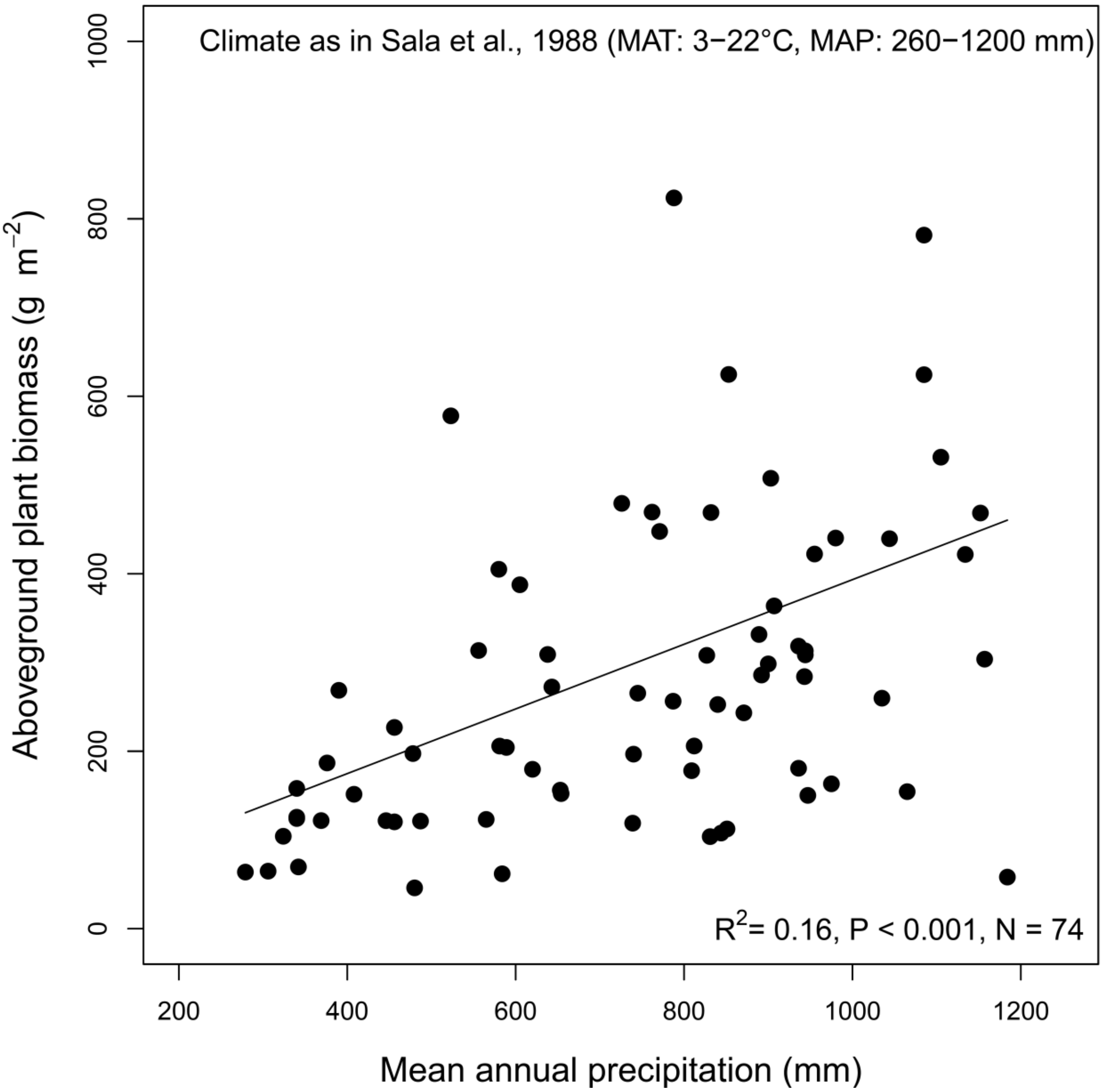

Figure S2 Aboveground plant biomass as a function of mean annual precipitation for a selection of sites. Only sites with MAT between 3 and 21°C and MAP between 260 and 1200 mm are included in this figure (N = 74). This selection of sites matches the one in Sala et al. (1988).

Table S1 Summary of models of log-transformed aboveground plant biomass (APB) with one or two quadratic terms, without and with multiplicative interactions. Shown are the P values of the models, the coefficients of determination (multiple and adjusted R^2) as well as the Akaike Information Criterion (AIC). For the models with interactions, the P value of the interactions of X_1 and X_2 as well as X_1^2 and X_2 is given. For each set of predictors, the best model (lowest AIC) is given in bold font. All models were calculated based on site-level data for all sites with data on soil clay content (N = 55). The independent variables (X) were centered, and APB was log-transformed (natural logarithm).

MAP: mean annual precipitation, MAT: mean annual temperature, TDry: temperature in the driest quarter of the year; Clay: soil clay content, N: soil total nitrogen content, Sha: Shannon index of plant diversity, rich: plant species richness, Simp: Simpsson index of plant diversity, NDep: atmospheric nitrogen deposition

X_1	X_2	Model structure	P value	P value interaction X_1 and X_2	P value interaction X_1^2 and X_2	Multiple R^2	Adj. R^2	AIC
MAP	MAT	$Y \sim X_1 + X_1^2 + X_2$	< 0.001	-	-	0.33	0.29	110
**MAP**	**MAT**	$\mathbf{Y \sim X_1{*}X_2 + X_1^2{*}X_2}$	**< 0.001**	**0.079**	**0.034**	**0.42**	**0.36**	**107**
MAP	MAT	$Y \sim X_1 + X_2 + X_1^2 + X_2^2$	< 0.001	-	-	0.33	0.28	112
MAP	MAT	$Y \sim X_1{*}X_2 + X_1^2{*}X_2 + X_1{*}X_2^2$	< 0.001	0.044	0.048	0.43	0.35	111
MAP	TDry	$Y \sim X_1 + X_1^2 + X_2$	< 0.001	-	-	0.37	0.34	106
MAP	TDry	$Y \sim X_1{*}X_2 + X_1^2{*}X_2$	< 0.001	0.127	0.492	0.40	0.34	108
**MAP**	**TDry**	$\mathbf{Y \sim X_1 + X_2 + X_1^2 + X_2^2}$	**< 0.001**	**-**	**-**	**0.41**	**0.36**	**105**
MAP	TDry	$Y \sim X_1{*}X_2 + X_1^2{*}X_2 + X_1{*}X_2^2$	< 0.001	0.192	0.771	0.43	0.34	112
**MAP**	**Clay**	$\mathbf{Y \sim X_1 + X_1^2 + X_2}$	**< 0.001**	**-**	**-**	**0.38**	**0.34**	**105**
MAP	Clay	$Y \sim X_1{*}X_2 + X_1^2{*}X_2$	< 0.001	0.528	0.980	0.38	0.32	110
MAP	Clay	$Y \sim X_1 + X_2 + X_1^2 + X_2^2$	< 0.001	-	-	0.38	0.33	108
MAP	Clay	$Y \sim X_1{*}X_2 + X_1^2{*}X_2 + X_1{*}X_2^2$	< 0.001	0.391	0.731	0.39	0.30	115
N	Sha	$Y \sim X_1 + X_1^2 + X_2$	< 0.001	-		0.30	0.26	112
**N**	**Sha**	$\mathbf{Y \sim X_1{*}X_2 + X_1^2{*}X_2}$	**< 0.001**	**0.027**	**0.025**	**0.38**	**0.32**	**110**
N	Sha	$Y \sim X_1 + X_2 + X_1^2 + X_2^2$	< 0.001	-		0.30	0.25	114
N	Sha	$Y \sim X_1{*}X_2 + X_1^2{*}X_2 + X_1{*}X_2^2$	< 0.001	0.168	0.097	0.42	0.33	112
N	rich	$Y \sim X_1 + X_1^2 + X_2$	< 0.001	-		0.31	0.27	130
**N**	**rich**	$\mathbf{Y \sim X_1{*}X_2 + X_1^2{*}X_2}$	**< 0.001**	**0.114**	**0.044**	**0.36**	**0.30**	**112**
N	rich	$Y \sim X_1 + X_2 + X_1^2 + X_2^2$	< 0.001	-		0.31	0.25	114
N	rich	$Y \sim X_1{*}X_2 + X_1^2{*}X_2 + X_1{*}X_2^2$	< 0.001	0.038	0.062	0.40	0.31	114

Table S1 continued

N	Simp	$Y\sim X_1 + X_1^2 + X_2$	< 0.001	-		0.30	0.26	112
**N**	**Simp**	**$Y\sim X_1*X_2 + X_1^2*X_2$**	**< 0.001**	**0.094**	**0.064**	**0.36**	**0.29**	**112**
N	Simp	$Y\sim X_1 + X_2 + X_1^2 + X_2^2$	< 0.001	-		0.31	0.25	114
N	Simp	$Y\sim X_1*X_2 + X_1^2*X_2+ X_1*X_2^2$	0.002	0.065	0.066	0.37	0.28	117
NDep	Sha	$Y\sim X_1 + X_1^2 + X_2$	0.006	-	-	0.21	0.17	118
**NDep**	**Sha**	**$Y\sim X_1*X_2 + X_1^2*X_2$**	**0.004**	**0.122**	**0.030**	**0.29**	**0.21**	**118**
NDep	Sha	$Y\sim X_1 + X_2 + X_1^2 + X_2^2$	0.009	-		0.23	0.17	119
NDep	Sha	$Y\sim X_1*X_2 + X_1^2*X_2+ X_1*X_2^2$	0.005	0.177	0.047	0.33	0.23	120
**NDep**	**rich**	**$Y\sim X_1 + X_1^2 + X_2$**	**0.006**	**-**	**-**	**0.22**	**0.17**	**118**
NDep	rich	$Y\sim X_1*X_2 + X_1^2*X_2$	0.007	0.629	0.096	0.27	0.20	119
NDep	rich	$Y\sim X_1 + X_2 + X_1^2 + X_2^2$	0.014	-		0.22	0.16	120
NDep	rich	$Y\sim X_1*X_2 + X_1^2*X_2+ X_1*X_2^2$	0.019	0.357	0.100	0.29	0.18	124
**NDep**	**Simp**	**$Y\sim X_1 + X_1^2 + X_2$**	**0.006**	**-**	**-**	**0.21**	**0.17**	**118**
NDep	Simp	$Y\sim X_1*X_2 + X_1^2*X_2$	0.009	0.390	0.080	0.26	0.19	120
NDep	Simp	$Y\sim X_1 + X_2 + X_1^2 + X_2^2$	0.014	-		0.22	0.15	121
NDep	Simp	$Y\sim X_1*X_2 + X_1^2*X_2+ X_1*X_2^2$	0.013	0.107	0.099	0.30	0.20	122

Table S2 Summary of models of aboveground plant biomass (APB) with one quadratic term and a multiplicative interactions. The models are the same as in Table S1 but here the APB was not log-transformed. Shown are the P values of the models, the coefficients of determination (multiple and adjusted R^2) as well as the Akaike Information Criterion (AIC). For the models with interactions, the P values of the interactions of X_1 and X_2 as well as X_1^2 and X_2 are given. All models were calculated based on site-level data for all sites with data on soil clay content (N = 55). The independent variables (X) were centered.

MAP: Mean annual precipitation, MAT: Mean annual temperature**,** N: soil total nitrogen content, Sha: Shannon index of plant diversity, NDep: atmospheric nitrogen deposition

X_1	X_2	Model structure	P value	P value interaction X_1 and X_2	P value interaction X_1^2 and X_2	Multiple R^2	Adj. R^2
MAP	MAT	$Y \sim X_1{*}X_2 + X_1^2{*}X_2$	0.006	0.051	0.029	0.28	0.20
N	Sha	$Y \sim X_1{*}X_2 + X_1^2{*}X_2$	< 0.001	0.004	0.011	0.43	0.38
NDep	Sha	$Y \sim X_1{*}X_2 + X_1^2{*}X_2$	0.005	0.006	0.007	0.28	0.21

Table S3 Author contributions according to the guidelines of the Nutrient Network.

Name	Developed and framed research question(s)	Analyzed data	Contributed to data analyses	Wrote the paper	Contributed to paper writing or editing	Site level coordinator	Nutrient Network Coordinators
Marie Spohn	×	×		×		×	
Sumanta Bagchi					×	×	
Jonathan D. Bakker					×	×	
Elizabeth T. Borer					×	×	×
Clinton Carbutt					×	×	
Jane A. Catford					×	×	
Christopher R. Dickman					×	×	
Nico Eisenhauer					×	×	
Anu Eskelinen					×	×	
Nicole Hagenah					×	×	
Yann Hautier					×	×	
Sally E. Koerner					×	×	
Kimberly J. Komatsu					×	×	
Lauri Laanisto					×	×	
Ylva Lekberg					×	×	
Jason P. Martina					×	×	
Holly Martinson					×	×	
Meelis Pärtel					×	×	
Pablo L. Peri					×	×	
Anita C. Risch					×	×	
Nicholas G. Smith					×	×	
Carly Stevens					×	×	
G.F. (Ciska) Veen					×	×	
Risto Virtanen					×	×	
Laura Yahdjian					×	×	
Alyssa L. Young					×	×	
Hillary S. Young					×	×	
Eric W. Seabloom					×	×	×
