# Supplementary material for: Interactive and unimodal relationships between plant biomass, abiotic factors, and plant diversity in global grasslands
Source: Commun Biol. 2025 Jan 21;8:97. doi: 10.1038/s42003-025-07518-w (PMC11751326; doi:10.1038/s42003-025-07518-w)
Supplement: Supplementary file 3 — Reporting summary [file 42003_2025_7518_MOESM3_ESM.pdf]

nature portfolio

Corresponding author(s): Marie Spohn

Last updated by author(s): 2024.12.17

Reporting Summary

Nature Portfolio wishes to improve the reproducibility of the work that we publish. This form provides structure for consistency and transparency in reporting. For further information on Nature Portfolio policies, see our Editorial Policies and the Editorial Policy Checklist.

Statistics

For all statistical analyses, confirm that the following items are present in the figure legend, table legend, main text, or Methods section.

n/a	Confirmed	
☐	☒	The exact sample size (*n*) for each experimental group/condition, given as a discrete number and unit of measurement
☐	☒	A statement on whether measurements were taken from distinct samples or whether the same sample was measured repeatedly
☐	☒	The statistical test(s) used AND whether they are one- or two-sided<br>*Only common tests should be described solely by name; describe more complex techniques in the Methods section.*
☐	☒	A description of all covariates tested
☐	☒	A description of any assumptions or corrections, such as tests of normality and adjustment for multiple comparisons
☐	☒	A full description of the statistical parameters including central tendency (e.g. means) or other basic estimates (e.g. regression coefficient) AND variation (e.g. standard deviation) or associated estimates of uncertainty (e.g. confidence intervals)
☐	☒	For null hypothesis testing, the test statistic (e.g. *F*, *t*, *r*) with confidence intervals, effect sizes, degrees of freedom and *P* value noted<br>*Give P values as exact values whenever suitable.*
☒	☐	For Bayesian analysis, information on the choice of priors and Markov chain Monte Carlo settings
☒	☐	For hierarchical and complex designs, identification of the appropriate level for tests and full reporting of outcomes
☒	☐	Estimates of effect sizes (e.g. Cohen's *d*, Pearson's *r*), indicating how they were calculated

Our web collection on statistics for biologists contains articles on many of the points above.

Software and code

Policy information about availability of computer code

Data collection	No code was used for data collection.
Data analysis	All data analyses were conducted using R (version 4.2.1).<br><br>We used the R packages piecewiseSEM (version 2.3.0), AICcmodavg (version 2.3-3), tidyverse (version 2.0), lattice (version 0.22-6)<br><br>All R code for reproducing the results is available at this repository.<br>https://doi.org/10.5281/zenodo.14509903

For manuscripts utilizing custom algorithms or software that are central to the research but not yet described in published literature, software must be made available to editors and reviewers. We strongly encourage code deposition in a community repository (e.g. GitHub). See the Nature Portfolio guidelines for submitting code & software for further information.

Data

Policy information about availability of data

All manuscripts must include a data availability statement. This statement should provide the following information, where applicable:

- Accession codes, unique identifiers, or web links for publicly available datasets
- A description of any restrictions on data availability
- For clinical datasets or third party data, please ensure that the statement adheres to our policy

All data are available at this repository.
https://doi.org/10.5281/zenodo.14509903

Research involving human participants, their data, or biological material

Policy information about studies with human participants or human data. See also policy information about sex, gender (identity/presentation), and sexual orientation and race, ethnicity and racism.

Reporting on sex and gender	not appplicabe
Reporting on race, ethnicity, or other socially relevant groupings	not appplicabe
Population characteristics	not appplicabe
Recruitment	not appplicabe
Ethics oversight	not appplicabe

Note that full information on the approval of the study protocol must also be provided in the manuscript.

Field-specific reporting

Please select the one below that is the best fit for your research. If you are not sure, read the appropriate sections before making your selection.

☐ Life sciences ☐ Behavioural & social sciences ☒ Ecological, evolutionary & environmental sciences

For a reference copy of the document with all sections, see nature.com/documents/nr-reporting-summary-flat.pdf

Ecological, evolutionary & environmental sciences study design

All studies must disclose on these points even when the disclosure is negative.

Study description	We collected standardized aboveground plant biomass (APB), plant diversity and environmental data at 116 natural and semi-natural grasslands on six continents (Fig. 1) that were not experimentally manipulated.
Research sample	All grassland sites studied here are natural or semi-natural grasslands that are part of the Nutrient Network Global Research Cooperative (Borer et al., 2014; https://nutnet.org). Mean annual precipitation at the sites varies between 192 to 1877 mm.
Sampling strategy	All sites followed the same sampling protocol, and the data were collected between 2007 and 2020. At each site, about 30 plots were established (range from 10 to 60 plots per site) that have a size of 5 × 5 m.
Data collection	Plant species composition was determined in a randomly designated 1×1m subplot within each 5×5m plot at peak biomass. In the 1 ×1m subplot, cover was estimated visually to the nearest 1% for every species overhanging the subplot.<br>Live vascular plant aboveground biomass (hereafter aboveground plant biomass or APB) was estimated at peak biomass (i.e., at the specific time of the year when aboveground plant biomass is largest). This was done destructively by clipping all aboveground biomass at ground level of plants rooted within two 1×0.1m strips (for a total of 0.2m2) adjacent to the 1×1m subplot where plant species composition was determined. All biomass was dried at 60°C to constant mass before weighing to the nearest 0.01g. Data on aboveground plant biomass and plant species composition were collected at all 116 sites.<br><br>Soil samples were collected in the 5 × 5 m plots by taking three soil cores (2.5 cm diameter) at a depth of 0 - 10 cm. The three cores were pooled to make one sample per plot. Root fragments were removed, and the soils were air-dried and sieved (< 2.0 mm) prior to any analysis. The samples were analyzed for total organic carbon and total nitrogen using an elemental analyzer (Costech ECS 4010 CHNSO Analyzer). Plant-available soil phosphorus (P), potassium (K), and calcium (Ca) were extracted from soil according to the Mehlich-3 protocol (Mehlich et al., 1984) and quantified using Inductively Coupled Plasma Mass Spectrometry. Soil texture, i.e., clay, silt and sand, was measured using the Bouyoucos method. All soil samples were analyzed in the same laboratory (Waypoint

	Analytical Laboratory, Memphis, Tennessee, USA). Data on soil chemical properties, including soil clay content, were collected for 55 of the 116 sites.
Timing and spatial scale	The 116 grasslands are located on six continents and samples were collected between 2007 and 2020.
Data exclusions	No data was excluded.
Reproducibility	All measurements were done according to the same protocol (Borer et al., 2014).
Randomization	There was no experimental treatment involved in this study.
Blinding	Sample identity and study design were not disclosed to technical personal involved in weighing biomass samples.

Did the study involve field work? ☒ Yes ☐ No

Field work, collection and transport

Field conditions	MAP at the sites varies between 192 to 1877 mm, while MAT varies between -6.6 and 27.3°C, soil clay content varies between 0.8 and 44.6%, and soil pH varies between pH 4.0 and 8.3.
Location	The 116 grasslands are located on six continents (See map in Fig. 1)
Access & import/export	Samples were collected from grassland sites located on research stations or land owned by universities and research institutes. Biomass was determined locally by local staff.
Disturbance	Disturbance in the field was minimized (by avoiding to step on the plots as much as possible).

Reporting for specific materials, systems and methods

We require information from authors about some types of materials, experimental systems and methods used in many studies. Here, indicate whether each material, system or method listed is relevant to your study. If you are not sure if a list item applies to your research, read the appropriate section before selecting a response.

Materials & experimental systems

n/a	Involved in the study	
☒	☐	Antibodies
☒	☐	Eukaryotic cell lines
☒	☐	Palaeontology and archaeology
☒	☐	Animals and other organisms
☒	☐	Clinical data
☒	☐	Dual use research of concern
☒	☐	Plants

Methods

n/a	Involved in the study	
☒	☐	ChIP-seq
☒	☐	Flow cytometry
☒	☐	MRI-based neuroimaging

Plants

Seed stocks	not appplicabe
Novel plant genotypes	not appplicabe
Authentication	not appplicabe
